# Photosensitive Organic-Inorganic Hybrid Materials for Room Temperature Gas Sensor Applications

**DOI:** 10.3390/nano8090671

**Published:** 2018-08-29

**Authors:** Marina Rumyantseva, Abulkosim Nasriddinov, Svetlana Vladimirova, Sergey Tokarev, Olga Fedorova, Ivan Krylov, Konstantin Drozdov, Alexander Baranchikov, Alexander Gaskov

**Affiliations:** 1 Chemistry Department, Moscow State University, 119991 Moscow, Russia; naf_1994@mail.ru (A.N.); vladimirova.lagytina@gmail.com (S.V.); pergeybokarev@gmail.com (S.T.); fedorova@ineos.ac.ru (O.F.); a.baranchikov@yandex.ru (A.B.); gaskov@inorg.chem.msu.ru (A.G.); 2 Nesmeyanov Institute of Organoelement Compounds of the Russian Academy of Sciences, 119991 Moscow, Russia; 3 Physics Department, Moscow State University, 119991 Moscow, Russia; ivan.phys@gmail.com (I.K); kadrozdov@gmail.com (K.D.); 4 Kurnakov Institute of General and Inorganic Chemistry of the Russian Academy of Sciences, 119991 Moscow, Russia

**Keywords:** organic–inorganic hybrid materials, tin dioxide, indium oxide, Ru(II) complex, NO_2_, semiconductor gas sensor, visible light activation

## Abstract

In this work, the hybrids based on nanocrystalline SnO_2_ or In_2_O_3_ semiconductor matrixes and heterocyclic Ru(II) complex are studied as materials for gas sensors operating at room temperature under photoactivation with visible light. Nanocrystalline semiconductor oxides are obtained by chemical precipitation with subsequent thermal annealing and characterized by XRD, SEM and single-point BET methods. The heterocyclic Ru(II) complex is synthesized for the first time and investigated by ^1^H NMR, ^13^C NMR APT, MALDI-MS analysis, and UV-Vis spectroscopy. The HOMO and LUMO energies of the Ru(II) complex are calculated from cyclic voltammetry data. The hybrid materials are characterized by TGA-MS analysis and EDX mapping. The optical properties of hybrids are studied by UV-Vis spectroscopy in the diffuse reflection mode. The investigation of spectral dependencies of photoconductivity of hybrid samples demonstrates that the role of organic dye consists in shifting the photosensitivity range towards longer wavelengths. Sensor measurements demonstrate that hybrid materials are able to detect NO_2_ in the concentration range of 0.25–2 ppm without the use of thermal heating under periodic illumination with even low-energy long-wavelength (red) light.

## 1. Introduction

The World Health Organization (WHO) has included inorganic gases, i.e., carbon monoxide (CO), nitrogen dioxide (NO_2_), sulphur dioxide (SO_2_), and ozone (O_3_), in the list of gases that have indoor sources, which are known in respect of their hazardousness to health and are often found indoors in concentrations of health concern [1]. Semiconductor gas sensors are promising for indoor and outdoor air monitoring because of their extremely high sensitivity, stability and miniaturization capability. The combination of gas sensors with information networks (including portable devices and mobile phones [2]) allows reporting a leakage and/or exceeding the maximum allowable concentration of hazardous gases upon short- or long-term exposures. However, the operating temperatures necessary for detection of the gases listed above are quite high: 250–350 °C (CO), 150–200 °C (NO_2_), 300–350 °C (SO_2_), 200–300 °C (O_3_) [3,4,5]. The need for heating significantly increases power consumption, which is the main restriction for coupling gas sensors with portable and mobile devices. Thus, the design of new materials that show gas sensitivity under conditions of minimal thermal heating is one of the key directions in developing the technology of gas sensors and multisensor systems.

When using semiconductor gas sensors, the formation of sensor response towards oxidizing gases (NO_2_, O_3_) occurs due to the adsorption of electron acceptor particles on the surface of semiconductors, which is accompanied by the localization of electrons on the adsorbed species. The recovery of the initial state of the surface and the electrophysical properties of the sensitive material occurs due to desorption of the molecules of the detected gas, which requires an increase in temperature. The use of photoactivation, instead of thermal heating, is an alternative, promising for lowering the power consumption of the sensor. The majority of articles that deal with sensor properties of semiconductor materials under illumination are devoted to studies carried out under UV light [6,7,8,9,10,11,12,13,14,15,16,17,18,19,20,21,22,23,24,25,26]. The light sources of the visible range (λ = 400–800 nm) have a lower energy consumption compared to the sources of UV radiation. In addition, the visible range corresponds to the maximum intensity of solar radiation, which can be additionally used as a source of illumination. However, bulk wide-gap metal oxides are transparent in the visible range of the spectrum. Based on the published data [27,28,29,30,31,32,33,34,35,36,37,38,39,40] for shifting the wavelength range of optical sensitivity of transparent conductive oxides towards longer wavelengths, it is necessary to create defects in the semiconductor matrix [27,28,29,30,31,32] or to introduce sensitizers, providing absorption in the visible region. Therefore, semiconductors of A^2^B^6^ group (CdS, *E_g_* = 2.4 eV; CdSe, *E_g_* = 1.7 eV) and A^3^B^5^ group (InP, *E_g_* = 1.35 eV) [28,33,34,35,36], as well as organic dyes (i.e., macrocyclic and heterocyclic complexes of transition metals [38,39,40]), are investigated.

Polypyridine Ru(II) complexes are intensively studied as photosensitizers for photochemical and photoelectrochemical conversion of solar energy [41]. The oxidation-reduction properties of the triplet metal-to-ligand charge transfer state in Ru(bpy_3_)^2+^ and its relatively long lifetime are the main reasons for its successful use in photovoltaic converters [42,43,44], and devices for water photolysis [43,44,45]. The possibility to adjust the optical and electrochemical properties by a reasonable choice of heterocyclic chelating ligands and relatively easy synthesis determines the suitability of using Ru (II) mononuclear complexes. The lowest unoccupied molecular orbitals (LUMOs) of diazine ligands with two-ring N-heteroatoms are lower in energy, compared with substituted bipyridines [46,47]. The possibilities of use of Ru (II) complexes with 3,3-bipyridazine [48], 2,2-bipyrazine [49], 2,2-bipyrimidine [50] and 4,4-bipyrimidine [51,52,53] ligands in devices for converting solar energy are discussed.

In the present research, the hybrids based on nanocrystalline SnO_2_ or In_2_O_3_ semiconductor matrixes and Ru(II) complex with 1*H*-imidazo[4,5-*f*][1,10]phenanthroline derivative were studied as materials for gas sensors operating at room temperature under photoactivation with visible light. Investigation of the interaction of hybrids with NO_2_ at room temperature under blue (λ_max_ = 470 nm), green (λ_max_ = 535 nm) and red (λ_max_ = 630 nm) light illumination has shown that the use of sensitization with organic dyes is an effective way to significantly improve the room-temperature gas sensor properties of semiconductor oxide materials.

## 2. Results and Discussion

### 2.1. Characteristics of Nanocrystalline Semiconductor Oxides

**Figure 1:**
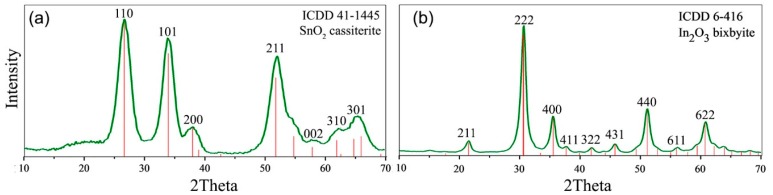
XRD patterns of SnO_2_ (**a**) and In_2_O_3_ (**b**) samples.

**Figure 2:**
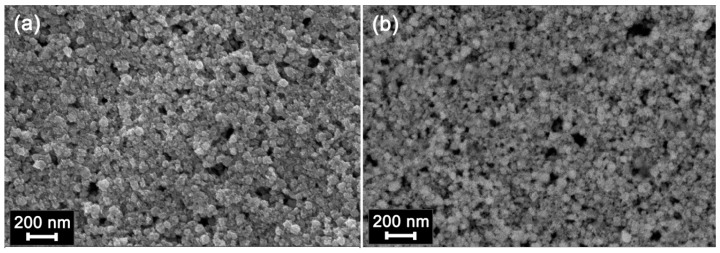
SEM images of In_2_O_3_ (**a**) and SnO_2_ (**b**) thick films deposited on functional substrates (see Materials and Methods) and sintered at 300 °C.

The diffraction pattern of SnO_2_ corresponds to the phase with the tetragonal cassiterite structure with a crystallite size of 3–4 nm, and the diffraction pattern of the In_2_O_3_ corresponds to the phase with a cubic structure of bixbyite with a crystallite size of 7–8 nm (Figure 1). The particle size distributions for SnO_2_ and In_2_O_3_ samples obtained under similar conditions were investigated by transmission electron microscopy (TEM) in our previous work [28]. The particle size determined by the TEM method was 4 ± 1 nm for SnO_2_ and 7 ± 2 nm for In_2_O_3_. The specific surface area determined by the single-point Brunauer-Emmett-Teller (BET) method was 110 ± 5 m^2^/g for SnO_2_ and 60 ± 5 m^2^/g for In_2_O_3_. Figure 2 presents the micrographs of In_2_O_3_ (a) and SnO_2_ (b) thick films formed on the dielectric substrate of the measured chip. Thick films were porous, consisting of agglomerated and sintered grains. The size of agglomerates was about 100 nm.

### 2.2. Characteristics of Ru(II) Heterocyclic Complex

**Figure 3:**
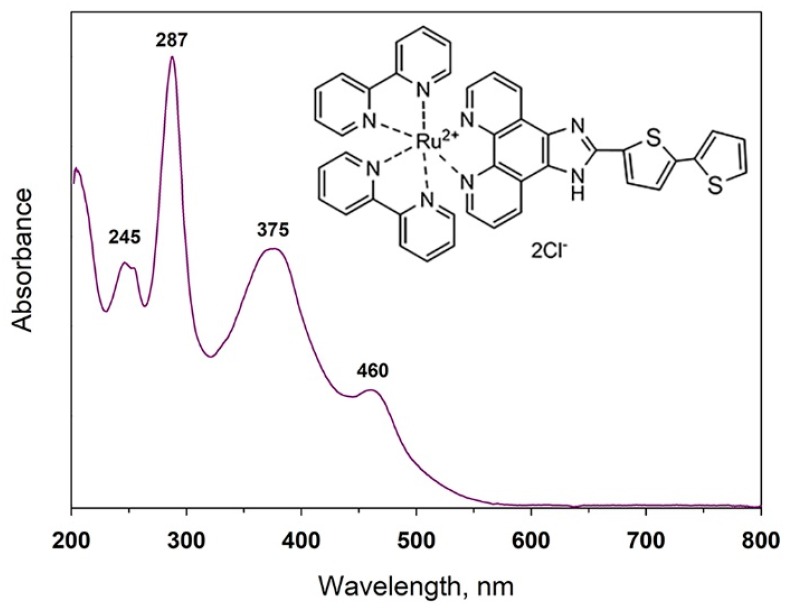
Structure and optical absorption spectrum of Ru-TT complex in a 5 × 10^−5^ M methanol solution.

The structure and absorption spectrum of heteroleptic Ru(II) complex bis(2,2′-bipyridine-k^2^*N*^1^,*N*^1^′)[2-(2,2′-bithiophen-5-yl)-1*H*-imidazo[4,5-*f*][1,10]phenanthroline-k^2^*N*^7^,*N*^8^) ruthenium(2^+^) dichloride (Ru-TT) are presented in Figure 3. The absorption bands in the UV range of Ru-TT (5 × 10^−5^ M solution in methanol) spectrum are due to ligand-centered (LC) π-π* transitions. The 1*H*-imidazo-phenantroline (ImPh) and pyridine π-π* transitions overlap in the 287 nm range [54,55]. Additionally, the π-π* transitions of substituted ImPh residues are in the region of 320–380 nm. In the visible region, Ru-TT complex displays the metal-to-ligand charge transfer (^1^MLCT) transition band (460 nm) associated with a d(Ru) -π* (substituted ImPh ligand) transition.

The voltammogram of Ru-TT complex exhibits three irreversible oxidation waves (***E*_1/2(*ox*)_**) and three reduction waves (***E*_1/2(red)_**) (Table 1). All observed electron reductions are ligand centered. The first two one-electron reductions of the complex are reversible, and the corresponding potentials are assigned to the reduction of the ImPh ligand as it is more easily reduced than 2,2-bipyridine. The third reduction potential at −1.96 V is very close to that obtained for the [Ru(bpy)_3_]^2+^ complex and is assigned to the reduction of the bpy ligand [56]. The oxidation potentials range from +1.23 V to +1.52 V for the heteroleptic complex and are attributed to the one-electron oxidation of the metal-centered highest-occupied molecular orbital (HOMO) (Table 1). The potential data for the metal-centered oxidation Ru(II/III), oxidation of ligands or chloride–anion should be close to each other, which makes interpretation of the obtained oxidation potential data difficult.

### 2.3. Characteristics of Hybrid Samples

**Figure 4:**
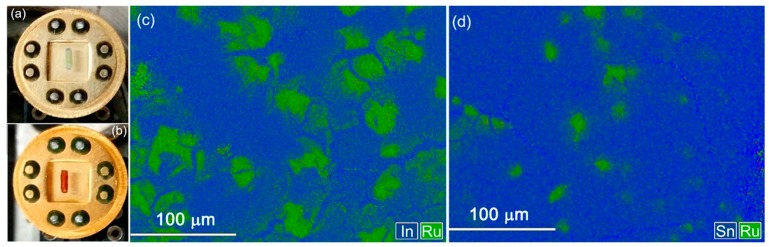
Optical images of SnO_2_ thick films deposited on functional substrates (see Materials and Methods) before (**a**) and after (**b**) sensitization with the Ru-TT complex. EDX maps of element distribution on the surface of In_2_O_3_ Ru-TT (**c**) and SnO_2_ Ru-TT (**d**) thick films.

The impregnation of SnO_2_ and In_2_O_3_ thick films with the Ru-TT methanolic solution resulted in appearance of bright red-orange coloration (Figure 4a,b). According to the results of energy-dispersive X-ray spectroscopy (EDX) mapping effectuated on sensitized thick films, the molecules of the heterocyclic Ru-TT complex were distributed unevenly on the surface of semiconductor oxides, filling regions with a diameter of 30–70 µm (Figure 4c,d). The average content of ruthenium in SnO_2_ and In_2_O_3_ thick films was $\frac{\left[Ru\right]}{\left[Ru\right]+\left[M\right]}$ = 1–2 at % (M = Sn, In) (Table 2).

**Figure 5:**
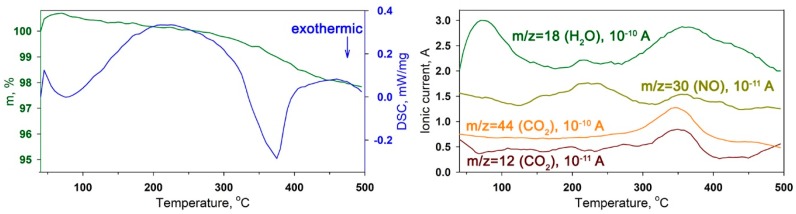
TG, DSC and MS curves of the SnO_2_ Ru-TT hybrid sample.

The range of thermal stability of semiconductor oxides sensitized with the Ru-TT complex was determined by thermogravimetric analysis combined with mass spectral analysis of gaseous products (TG-MS). Figure 5 shows the MS signals of CO_2_ (m/z = 12, m/z = 44), NO (m/z = 30), and H_2_O (m/z = 18), corresponding to the fragmentation products of the SnO_2_ Ru-TT sample. Analysis of thermograms and mass spectra showed that, in the temperature range 35–150 °C, the sample was dried with removal of adsorbed water. The decomposition of Ru-TT complex was exothermic and set on above 200 °C, as evidenced by a pronounced peak on the curve of thermal effects and CO_2_ emission. Thus, under conditions of sensor measurements at room temperature, the sensitizer was thermally stable.

**Figure 6:**
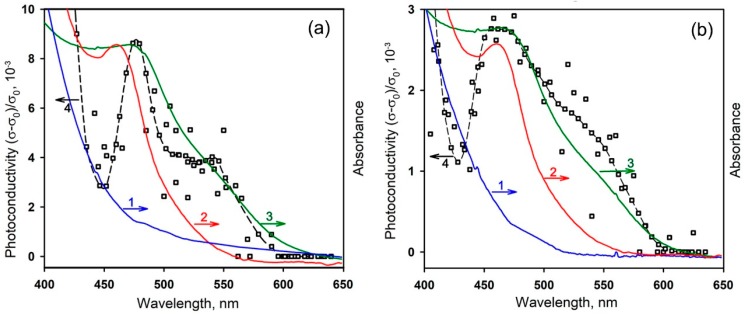
Absorption spectra of the pure semiconductor oxide (1), Ru-TT complex (2), hybrid sample (3) and spectral dependences of photoconductivity (4) of In_2_O_3_ Ru-TT (**a**) and SnO_2_ Ru-TT (**b**).

A comparison of optical and photoelectrical properties of pure semiconductor matrixes, Ru-TT complex and hybrid materials is shown in Figure 6. The greatest influence of Ru-TT complex on the optical properties of composites appeared in visible spectral range. In both hybrids, the intensive absorption band was observed at λ_max_ = 472 nm, due to the presence of organic sensitizer. As compared with the spectrum of Ru-TT complex, the maximum of the absorption edge was shifted to the long-wavelength region by about 10 nm. Even though the constituent components of hybrids do not have absorption bands at λ > 500 nm, the spectra of sensitized materials exhibited a broad shoulder in the long-wavelength region around 510–620 nm. This can be caused by the aggregation of Ru-TT molecules on the surface of the semiconductor oxides [57].

The photoconductivity of hybrid samples (Figure 6) in the visible spectral region increased from 600 nm and reached a maximum value at 480–500 nm. The shape of the spectral dependences of the hybrid’s photoconductivity agrees with their optical absorption spectra. This allows us to state that the photoexcitation of organic dye is accompanied by the injection of electrons from the Ru-TT complex into In_2_O_3_ and SnO_2_ semiconductor matrixes.

### 2.4. Gas Sensor Properties

**Figure 7:**
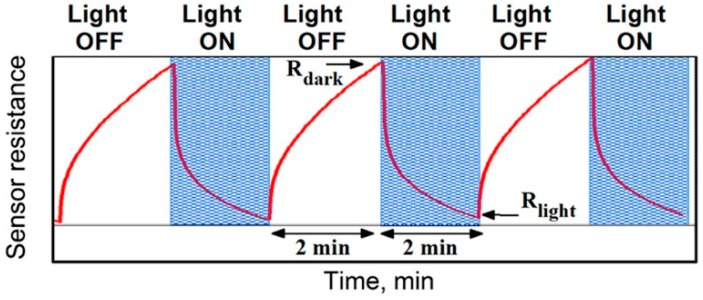
Scheme of resistance change of n-type semiconductor under periodic illumination.

Our previous investigations of gas sensor properties of nanocomposites, consisting of a semiconductor oxide matrix and photosensitizers—semiconductor quantum dots, performed under constant and periodic illumination, demonstrated that the latter option is preferable because of long recovery time constants (more than 1 h) necessary to return to the equilibrium resistance under constant illumination [35,58]. In this procedure, the illumination of the sensor element is carried out in a pulsed mode with a short period (2 min “on”–2 min “off”). If the illumination cycle is repeated, the change in the sensor resistance in each of the following cycles comes very close to the previous one. This steady state can be characterized by the minimum resistance *R_light_*, which is achieved during the sensor illumination, and the maximum *R_dark_*, which is achieved in the dark period (Figure 7). From these values, the effective photoresponse can be calculated as:

(1) $$S_{Ph}=\frac{R_{dark}}{R_{light}}.$$

**Figure 8:**
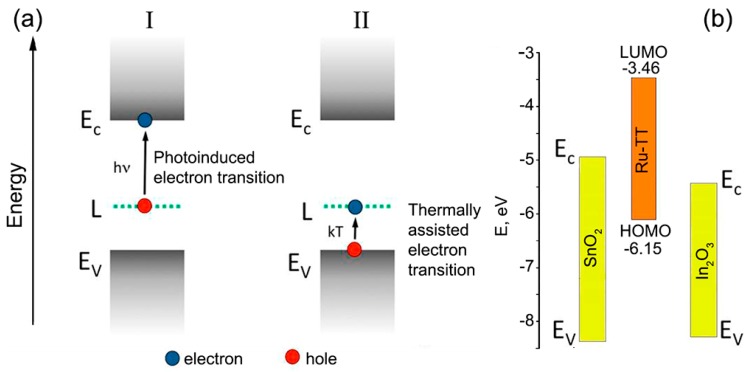
(**a**) Scheme of the consequential mechanism for the generation of holes in pure nanocrystalline oxides under visible light illumination. *E_v_*—valence band, *E_c_*—conduction band, *L*—acceptor level. (**b**) Scheme of the mutual arrangement of the energy levels for bulk In_2_O_3_, SnO_2_, and HOMO and LUMO of the Ru-TT complex.

In pure air, the decrease of the material resistance under illumination and its increase upon turning the light off are determined by the dynamic equilibrium between adsorption and photodesorption of oxygen:

(2) $$O_{2\left(gas\right)}+e^{-}↔O_{2\left(ads\right)}^{-},$$

(3) $$O_{2\left(ads\right)}^{-}+h^{+}↔O_{2\left(gas\right)},$$

where $O_{2\left(gas\right)}$ is an oxygen molecule in the gas phase, $O_{2\left(ads\right)}^{-}$ is the molecular form of chemisorbed oxygen, $e^{-}$ is an electron, which can be localized on an electrophilic oxygen molecule, and $h^{+}$ is a photogenerated hole. For bulk wide-gap semiconductor oxides, the photodesorption of oxygen occurs only under UV illumination. However, for nanocrystalline materials, this process becomes possible even under visible light due to the presence of sub-band surface states [59]. In this case, the consequential mechanism for the generation of holes is realized: (I) under visible light, the electrons localized at acceptor levels in the forbidden band of semiconductor are transferred to the conduction band, (II) thermal transitions of electrons from the valence band to empty acceptor levels result in hole generation (Figure 8a). This mechanism is much weaker than the band gap mechanism realized under UV light, and the corresponding threshold energy is *E_g_* − *E_a_*, where *E_a_* is the ionization energy of the acceptor [60]. The small value of the visible light photoresponse observed for nanocrystalline oxides SnO_2_ and In_2_O_3_ in air (Table 2) confirms the realization of this mechanism.

The introduction of an organic sensitizer leads to an increase in the photoresponse in pure air of both tin oxide and indium oxide (Table 2). Figure 8b illustrates the mutual arrangement of the energy levels for bulk In_2_O_3_, SnO_2_, and HOMO and LUMO of the Ru-TT complex. The positions of valence (*E_V_*) and conduction (*E_C_*) bands for bulk SnO_2_ and In_2_O_3_ were taken from ref. [61]. When hybrids are illuminated with visible light, the electrons of Ru-TT complex are excited from the HOMO to LUMO level, which is above the conduction band minimum of semiconductor matrix. These photoexcited electrons can be transferred to the conduction bands of SnO_2_ and In_2_O_3_, which results in an increase in their conductivity. Therefore, under illumination, the HOMO level of organic complex becomes electron-depleted. To regenerate the dye, it is necessary to ensure the transfer of the electron from any reducing particle to its HOMO. This can be done by the electrons localized by chemisorbed oxygen on the surface of the semiconductor oxide. Therefore, the oxygen molecule passes back into a physically adsorbed neutral form, which can be easily removed from the surface.

Since nitrogen dioxide (electron affinity 2.27 eV [62]) is a stronger electron acceptor than oxygen (electron affinity 0.44 eV [63]), the adsorption equilibria:

(4) $$NO_{2\left(gas\right)}+e^{-}↔NO_{2\left(ads\right)}^{-}$$

(5) $$NO_{2\left(gas\right)}+O_{2\left(ads\right)}^{-}↔NO_{2\left(ads\right)}^{-}+O_{2\left(gas\right)}$$

are more strongly shifted towards the chemisorbed form of NO_2_ as compared to the chemisorbed form of O_2_.

It has been shown in [12,35,64] that, under dark conditions, the adsorption of NO_2_ on the surface of semiconductor oxides proceeds irreversibly at room temperature. Thus, when NO_2_ is removed from the atmosphere, its desorption from the surface of the semiconductor oxide does not occur. However, this desorption process is a prerequisite for restoring the initial state of the surface and the electrical properties of the sensitive material, providing the formation of a reproducible sensor signal. In our previous works [28,35,58], it was demonstrated that the role of illumination consists in the photogeneration of holes that ensure (as in the case of oxygen) the conversion of chemisorbed particles into physically sorbed ones, which can be easily desorbed from the surface of the semiconductor oxide even at room temperature due to thermal fluctuations:

(6) $$NO_{2\left(ads\right)}^{-}+h^{+}↔NO_{2\left(gas\right)}$$

**Figure 9:**
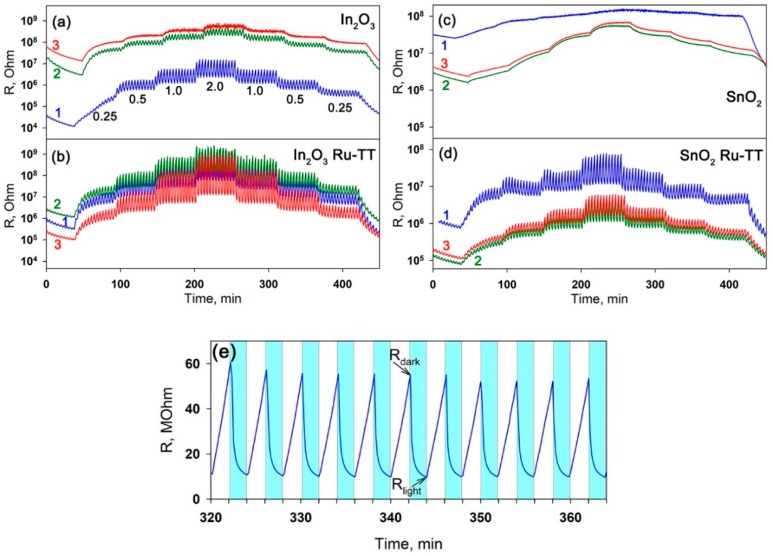
Room-temperature electrical resistances of (**a**) In_2_O_3_, (**b**) In_2_O_3_ Ru-TT, (**c**) SnO_2_, and (**d**) SnO_2_ Ru-TT samples under periodic illumination, depending on NO_2_ content in the gas phase. (1): blue light (λ_max_ = 470 nm), (2): green light (λ_max_ = 535 nm), (3): red light (λ_max_ = 630 nm). The digits on Figure 9a show the sequence of changes in NO_2_ concentration (ppm). (**e**) Room-temperature electrical resistance of the In_2_O_3_ Ru-TT sample in the presence of the 0.5-ppm NO_2_ concentration under periodic blue light (λ_max_ = 470 nm) illumination. The shaded areas correspond to the “light on” period.

The investigation of sensor properties of pure nanocrystalline oxides and hybrids in NO_2_ detection were performed with periodic illumination at room temperature. The concentration of NO_2_ was changed stepwise, first in the direction of increasing concentration, and then in descending order (Figure 9). With the growth of NO_2_ concentration, the resistance values of *R_light_* and *R_dark_* increased for all samples (Figure 9) in accordance with the dynamic equilibrium between the processes of NO_2_ adsorption (Equations (4) and (5)) and photodesorption (Equation (6)).

The amplitude of effective photoresponse (resistance change) at a fixed NO_2_ concentration is nearly constant (Figure 9e). Hence, the sensor signal can be calculated [35] as:

(7) $$S=\frac{R_{dark}}{R_{dark0}},$$

where *R_dark_* is the dark resistance (measured at the end of the 2-min “light off” period, Figure 7) at a given NO_2_ concentration and *R_dark_*_0_ is the dark resistance in pure air. This method of sensor signal calculation is preferred, since the adsorption of NO_2_ takes place to the greatest extent under the dark conditions.

**Figure 10:**
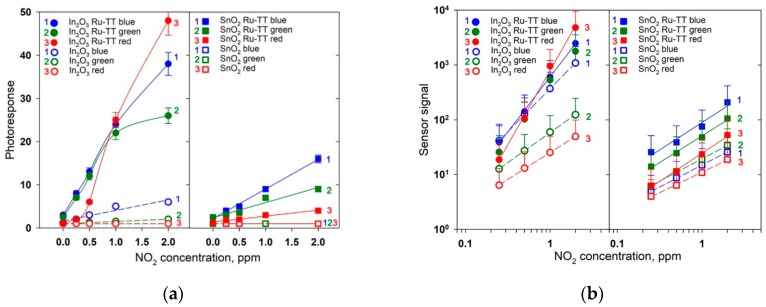
Effective room-temperature photoresponse (**a**) and sensor signal (**b**) of In_2_O_3_- and SnO_2_-based samples under periodic visible light illumination, depending on NO_2_ content in the gas phase. Open symbols: data for blank matrixes, filed symbols: data for hybrid materials. (1): blue light (λ_max_ = 470 nm), (2): green light (λ_max_ = 535 nm), (3): red light (λ_max_ = 630 nm).

Figure 10 compares the dependencies of the effective photoresponse and sensor signal on the concentration of NO_2_ in air for pure semiconductor oxides and hybrid materials under illumination with blue, green and red light.

The main trends can be summarized as follows.

(i) Pure tin dioxide did not exhibit photosensitivity (Figure 9c), and the effective photoresponse *S_Ph_* = 1 for all NO_2_ concentrations under illumination with blue, green and red light (Figure 10a). Nevertheless, the observed change in the resistance as a function of the NO_2_ concentration makes it possible to calculate the magnitude of the sensor signal by Equation (7). The maximum sensor signal of blank SnO_2_ was measured under green light illumination (Figure 10b).
  This effect can be due to the participation of oxygen vacancies of tin dioxide in the adsorption of NO_2_. As shown by the authors of [65], the acceptor levels related to the oxygen vacancies in SnO_2_ lie at 1.4 eV (bridging vacancies) and 0.9 eV (in-plane vacancies) above the valence band, which correspond to the energy of an electron transition from an acceptor level of *E_a_* = 2.2 eV (563 nm) and *E_a_* = 2.3 eV (538 nm), respectively. The absence of the photosensitivity (*S_Ph_* = 1), together with the measurable sensor signal (*S* > 1), can be due to the fact that, for the finely dispersed tin dioxide, the main process of interaction with NO_2_ is the reaction (5). Since the electron affinity of NO_2_ is larger than the one for O_2_, the position of energy levels of electrons localized on NO_2_ molecules chemisorbed on a SnO_2_ surface is deeper than that for chemisorbed oxygen. Thus, electron transfer in accordance with the reaction (5) will lead to a decrease in the Fermi level of the semiconductor. Since the band structure of nanocrystalline SnO_2_ with a particle size of 3–4 nm corresponds to the situation of flat zones [66], the decrease in the Fermi level indicates the decrease in the electrical conductivity, providing the sensor signal in the presence of NO_2_.
(ii) With a blue illumination, pure In_2_O_3_ exhibited a noticeable photosensitivity (Figure 9a). The value of the effective photoresponse at a fixed concentration of NO_2_ decreased with the increasing wavelength of the activating light (wavelengths of 470, 535 and 630 nm were used) (Figure 10a). A similar trend was observed for the concentration dependence of sensor signal. The maximum values of sensor signal in the whole range of NO_2_ concentrations were obtained under blue light (Figure 10b). This tendency is in accordance with the fact that for nanocrystalline In_2_O_3_, the photoconductivity is nonzero at photon energies more than 2.25 eV (λ < 550 nm), which can be explained by the generation of electrons from localized levels located in the bandgap [64].
(iii) The sensitization of semiconducting oxides with the Ru-TT organic complex leads to the increase in both the effective photoresponse *S_Ph_* of the materials and their sensor signal *S* towards NO_2_ (Figure 9b,d). For the SnO_2_ R-TT hybrid material, the effective photoresponse and sensor signal values decreased upon transition from blue to green and then to red light illumination (Figure 10). The observed tendency agrees with the absorption spectrum and the spectral dependence of the photoconductivity of this hybrid material. In the case of the In_2_O_3_ Ru-TT hybrid, the maximum values of the photoresponse and the sensor signal at high concentrations of NO_2_ were obtained under red light. It appears to be an artifact. The values of the dark resistance *R_dark_* observed under these measurement conditions exceeded 10^9^ Ohm, which is the upper limit of the measuring system. The measurement of such resistances occurred with a large error and a high noise level.

## 3. Materials and Methods

### 3.1. Materials Synthesis

#### 3.1.1. Synthesis of Ru(II) Complex

**Figure 11:**
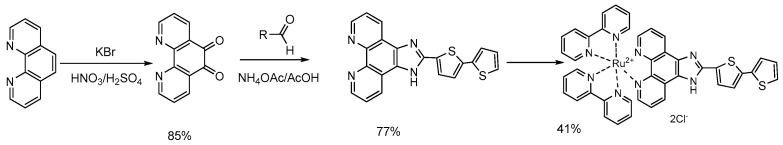
Scheme of the synthesis of Ru-TT complex.

The synthesis of heteroleptic Ru(II) complex bis(2,2′-bipyridine-k^2^*N*^1^,*N*^1^′) [2-(2,2′-bithiophen-5-yl)-1*H*-imidazo[4,5-*f*][1,10]phenanthroline-k^2^*N*^7^,*N*^8^) ruthenium(2+) dichloride (Ru-TT) was carried out according to the scheme presented in Figure 11. The first stage was the oxidation of 1,10-phenanthroline with KBrO_3_ to provide phenetroline-6,10-dione [67]. Condensation reactions of the aldehydes with phenetroline-6,10-dione gave the ligand as a bright crystalline compound with good yield of 77% [68,69]. To prepare the Ru-TT complex, an equimolar mixture of the ligand with Ru(bpy)_2_Cl_2_ (*cis*-bis(2,2-bipyridine)dichlororuthenium II hydrate) was kept in ethanol at 80 °C in sealed ampoule in argon for 8 h [70]. After complete reaction, the crude complex with a yield of 41% was purified by column chromatography. 

The structure of the product was determined by ^1^H NMR, ^13^C NMR APT and MALDI-MS analysis.

^1^H NMR (methanol-d4, δ; ppm, J/Hz): 7.08 (t, 1H, ^3^*J* = 4.3, Th(4′)), 7.22 (d, 1H, ^3^*J* = 3.8, Th(3′)), 7.26 (d, 1H, ^3^*J* =3.5, Th(3)), 7.37 (m, 3H, Th(5′), Py(5′)), 7.57 (t, 2H, ^3^*J* = 6.6, Py(5)), 7.71 (dd, 2H, ^3^*J* = 5.3, ^3^*J* = 8.2, ImPh(5,10)), 7.74 (d, 2H, ^3^*J* = 5.7, Py(6′)), 7,81 (d, 1H, ^3^*J* = 3.7, Th(4)), 7.97 (m, 4H, ImPh(6,9), Py(6)), 8.07 (t, 2H, ^3^*J* = 7.9, Py(4′)), 8.18 (t, 2H, ^3^*J* = 7.9, Py(4)), 8.74 (d, 2H, ^3^*J* = 8.1, Py(3′)), 8.77 (d, 2H, ^3^*J* = 8.2, Py(3)), 8.99 (d, 2H, ^3^*J* = 8.2, ImPh(4,11).

^13^C NMR APT (methanol-d4, δ; ppm, J/Hz): 123.93, 124.24, 124.35, 125.00, 125.13, 127.02, 127.53, 127.57, 127.87, 130.25, 137.71, 137.79, 148.52, 151.43, 151.60 [CH], 124.31, 134.30, 134.62, 136.37, 138.81, 150.93, 144.94, 157.20, 157.42 [C].

MALDI-MS m/z: 398 [M-2Cl]^2+^.

The chemical composition of the product ((%) C, 56.78; H, 3.24; N, 12.98; Ru, 11.56) agrees well with the value calculated for C_41_H_28_Cl_2_N_8_RuS_2_ ((%): C, 56.68; H, 3.25; N, 12.90; Ru, 11.63).

#### 3.1.2. Synthesis of Nanocrystalline SnO_2_ and In_2_O_3_ and Hybrid Materials

The powders of nanocrystalline SnO_2_ and In_2_O_3_ were prepared, from SnCl_4_*5H_2_O and In(NO_3_)_3_·4.5H_2_O, respectively, by chemical precipitation method as described in [28]. After the stages of washing and drying, the products were annealed in air at 300 °C for 24 h.

To study the thermal stability and optical properties, hybrids based on the semiconductor oxides and Ru-TT complex were prepared in the form of powders. Firstly, Ru-TT was dissolved in methanol; then, 10 μL of the obtained solution was added dropwise to the weighed sample of the semiconductor oxide and the paste was dried until the solvent was completely evaporated. The concentration of the solution was selected, so that the Ru content in the hybrids was 1 wt. %.

**Figure 12:**
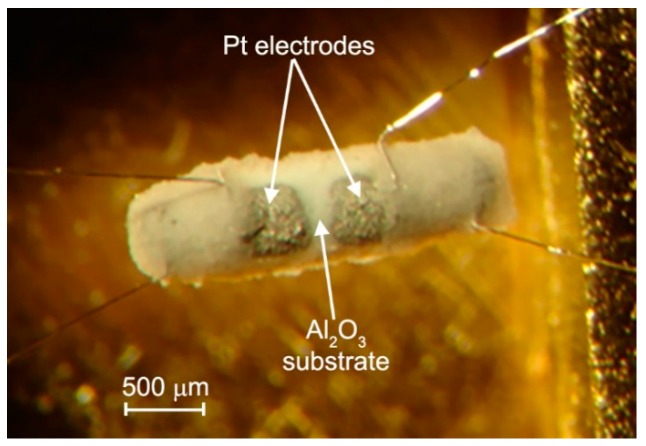
Optical image (top view) of the Al_2_O_3_ substrate with Pt electrodes. The Pt heather is on the back side (not shown).

To investigate the spectral dependence of photoconductivity and gas sensor properties, the hybrid materials were prepared in the form of thick films on alumina substrates with Pt electrodes on the top side and Pt microheater on the back side (Figure 12). The powders of SnO_2_ and In_2_O_3_ (~10 mg) were mixed with a binder (α-terpineol in ethanol). The obtained pastes were deposited on the substrates by the micro dropping technique, dried at 30 °C for 24 h in air and sintered at 300 °C for 10 h in air using Pt microheaters. Then, the films were impregnated with the calculated amount of methanolic Ru-TT solution and dried at 50 °C for 24 h. The estimated film thickness was about 2–3 µm.

### 3.2. Materials Characterization

The phase composition was examined by X-ray powder diffraction (XRD) with D/MAX-2500V/PC diffractometer (Rigaku, Tokyo, Japan) (λ = 1.54059 Å (Cu K_α1_ radiation)). The crystallite sizes of SnO_2_ and In_2_O_3_ were calculated from the broadening of the most intensive XRD peaks using the Scherer equation. The single-point BET surface area of nanocrystalline oxides was determined using Chemisorb 2750 (Micromeritics, Norcross, GA, USA). The microstructure and composition of hybrid thick films were investigated directly on functional substrates (Figure 12) by scanning electron microscopy (SEM) combined with EDX at Zeiss NVision 40 (Carl Zeiss, Oberkochen, Germany) microscope equipped with an X-Max detector (Oxford Instruments, Abingdon, UK).

The thermal stability of hybrid materials was investigated by thermogravimetric analysis combined with mass spectroscopy analysis of gaseous products (TG-MS) using a thermal analyzer STA 409 PC Luxx (Netzsch-Gerätebau GmbH, Selb, Germany) with a quadrupole mass spectrometer QMS 403 C Aëolos (Netzsch-Gerätebau GmbH, Selb, Germany). The powders of hybrid materials were heated up to 500 °C with a heating rate of 10 °C/min in air.

The absorption spectrum of Ru-TT complex was measured with a Varian Cary 50 spectrometer (Varian Inc., Palo Alto, CA, USA) in the 200–800 nm range in 1 nm increments using a quartz cuvette with an optical path length of 10 mm. The absorption spectra of semiconductor oxides and hybrid powders were obtained with a Perkin-Elmer Lambda-950 spectrometer (PerkinElmer Inc., Waltham, MA, USA) in the diffuse reflection mode in the 200–800 nm range in 1 nm increments with a preliminary subtraction of the baseline.

The electrochemical characterization of the Ru-TT complex was carried out at 22 °C with an IPC-Pro M potentiostat (Volta, St. Petersburg, Russia). Cyclic voltammetry experiments were performed in a 1.0 mL cell equipped with a glassy carbon (GC) electrode (disk *d* = 2 mm), Ag/AgCl/KCl (aq. saturated; reference electrode), and platinum electrode (counter electrode). The complexes were dissolved in degassed dry CH_3_CN containing *tetra*-(butyl)ammonium perchlorate (TBAP) as the supporting electrolyte (0.10 M). Dry argon gas was bubbled through the solutions for 30 min before cyclic voltammetry experiments. The scan rate was 200 mVs^−1^. A 10^−3^ M solution of ferrocene with 0.1 M TBAP in the same solvents was used for calibration.

The HOMO and LUMO energies of the Ru-TT complex were calculated using the methodology described in [71], using the following equations:

*E*_HOMO_ = −4.73 − *E*_onset (Ox);_ (8)

*E*_LUMO_ = −4.73 − *E*_onset (Red)_ (9)

Spectral dependence of photoconductivity of hybrid materials was investigated in a cell shielded from the background light. Using a combination of the white light output of 100 W/cm^2^ and an MDR-206 monochromator (LOMO Photonica, St. Petersburg, Russia), the samples were illuminated for 20 s at each wavelength with 5 nm increments. The dark interval between the measurements was 60 min. The photoconductivity was calculated as a conductance ratio:

(10) $$\frac{\Delta \sigma \;}{\sigma_{0}}=\frac{\sigma \left(\lambda \right)-\sigma_{0}}{\sigma_{0}}$$

where *σ*(*λ*) is the film conductance under illumination with corresponding λ, *σ*_0_ is the film conductance under dark conditions.

All sensor measurements were carried out at room temperature in the flow cell shielded from the background light under a controlled constant gas flux of 100 mL/min. DC measurements (U = 3 V) were carried out to monitor the electrical conductance of the sample during exposure to NO_2_/air gas mixtures (0.25–2.0 ppm NO_2_ in dry air). Miniature light emission diodes (LEDs) with λ_max_ = 470 nm (blue), λ_max_ = 535 nm (green) and λ_max_ = 630 nm (red) inserted into the cell were used as illumination sources.

## 4. Conclusions

It was demonstrated that the use of the heterocyclic Ru(II) complex (Ru-TT) as a photosensitizer results in a shift of the photosensitivity of the wide-gap nanocrystalline metal oxides SnO_2_ and In_2_O_3_ toward longer wavelengths. The creation of hybrid materials not only leads to an enhanced absorbance of nanocrystalline oxides in the visible range of the spectrum, but also expands the absorption region of the sensitized material, compared to the original organic dye. The shape of the spectral dependences of the hybrid’s photoconductivity agrees with their optical absorption spectra. This indicates that the photoexcitation of organic dye is accompanied by the injection of electrons from the Ru-TT complex into In_2_O_3_ and SnO_2_ semiconductor matrixes. The sensitization of semiconducting oxides with the organic dye leads to the increase in both the effective photoresponse *S_Ph_* of the materials and their sensor signal *S* towards NO_2_. The ratios of the sensor signal to 2 ppm of NO_2_ for sensitized and blank matrixes described for In_2_O_3_-based samples as $\frac{S_{In2O3-RuTT}}{S_{In2O3}}$, were 2.5, 10, and 10^2^ for the activating light wavelength at 470, 535 and 630 nm, respectively, and for the SnO_2_-based samples, written as $\frac{S_{SnO2-RuTT}}{S_{SnO2}}$, were approximately 8, 3, 3 under the illumination of the activating light at 470, 535 and 630 nm, respectively. Hybrid materials are capable to detect NO_2_ in the concentration range of 0.25–2 ppm at room temperature (without the use of thermal heating) under periodic illumination with low-energy long-wavelength (red) light. From the point of view of increasing the sensor signal, the sensitization by the Ru-TT organic complex is more effective in cases where the semiconductor matrix is weakly sensitive to the radiation used.

## Figures and Tables

**Table 1:** Electrochemical data and calculated values of HOMO and LUMO of Ru-TT complex.

*E*_1/2(red)_, V	*E*_1/2(ox)_, V	*E*_HOMO_, eV	*E*_LUMO_, eV
−1.27/−1.21	1.23	−6.15	−3.46
−1.48/−1.41	1.42		
−1.96/−1.84	1.52		

**Table 2:** Microstructure characteristics, composition and photoresponse of investigated samples.

Sample	d_XRD_^a^, nm	d_TEM_^b^, nm	S_surf_^c^, m^2^/g	$\frac{\left[Ru\right]}{\left[Ru\right]+\left[M\right]{\;}^{d}},\;at.\%$	S_Ph_^e^ in Pure Air (λ = 470 nm)
SnO_2_	4 ± 1	4 ± 1	110 ± 5	-	1.00
SnO_2_ Ru-TT				1.4 ± 0.1	2.72
In_2_O_3_	7 ± 1	7 ± 2	60 ± 5	-	1.30
In_2_O_3_ Ru-TT				2.1 ± 0.2	3.15

^a^ MO_x_ crystallite size, estimated from XRD data; ^b^ MO_x_ particle size (TEM); ^c^ MO_x_ specific surface area; ^d^ obtained by EDX on thick films: M = Sn for the SnO_2_ Ru-TT sample; M = In for the In_2_O_3_ Ru-TT sample; ^e^ Effective photoresponse.
